# The contribution of major histocompatibility complex contacts to the affinity and kinetics of T cell receptor binding

**DOI:** 10.1038/srep35326

**Published:** 2016-10-13

**Authors:** Hao Zhang, Hong-Sheng Lim, Berhard Knapp, Charlotte M. Deane, Milos Aleksic, Omer Dushek, P. Anton van der Merwe

**Affiliations:** 1 Sir William Dunn School of Pathology, University of Oxford, United Kingdom; 2 Department of Statistics, University of Oxford, United Kingdom

## Abstract

The interaction between the T cell antigen receptor (TCR) and antigenic peptide in complex with major histocompatibility complex (MHC) molecules is a crucial step in T cell activation. The relative contributions of TCR:peptide and TCR:MHC contacts to the overall binding energy remain unclear. This has important implications for our understanding of T cell development and function. In this study we used site directed mutagenesis to estimate the contribution of HLA-A2 side-chains to the binding of four TCRs. Our results show that these TCRs have very different energetic ‘footprints’ on HLA-A2, with no residues contributing to all TCR interactions. The estimated overall contribution of MHC side-chains to the total interaction energy was variable, with lower limits ranging from 11% to 50%. Kinetic analysis suggested a minor and variable contribution of MHC side-chains to the transition state complex, arguing against a two-step mechanism for TCR binding.

A key event in the adaptive immune response is antigen recognition by T cells, which is required for T cell activation, differentiation and effector function. Antigen recognition by conventional T cells typically requires the binding of the TCR to a complex between a peptide antigen and an MHC molecule on the surface of an antigen presenting cell. Because of its crucial importance, the interaction between TCR and peptide-MHC (pMHC) has been extensively studied, providing important insights into the structure and binding properties of TCR-pMHC interactions [reviewed in refs 1-8].

Unsurprisingly, given the huge diversity in TCRs, peptide antigens, and MHC molecules, there is considerable variability in the fine structure of the TCR/pMHC interface. However, some features are conserved. Firstly, TCRs make contact via their variable complementarity determining region (CDR) loops with both the peptide and the MHC, with CDR3 loops positioned over the peptide at the center of the interface. Secondly, the binding orientation is broadly diagonal, with the TCR Vα CDR loops typically positioned over the N-terminal end of the peptide and/or the α2 helix of MHC class I (or the equivalent helix of MHC II). Finally, the proportion of the contact interface contributed by TCR/peptide contacts is lower (20–40%) than the portion contributed by TCR/MHC contacts (60–80%)^1^,^8^.

Although structural studies can accurately identify physical contacts at a binding interface, termed the ‘structural footprint’, they do not reveal to what extent these contacts contribute to the binding energy or affinity of that interaction, termed the ‘energetic footprint’^9^, and it is the latter that is likely to be functionally important. The relative contribution of TCR/peptide and TCR/MHC contacts to the energetic footprint of TCR/pMHC interactions has important functional implications. If TCR/MHC contacts are dominant this could make TCR recognition less dependent on the peptide sequence, potentially increasing the promiscuity of the TCR and the likelihood of autoimmunity. Conversely, if TCR/peptide contacts are dominant then any given TCR may only be able to recognise a relatively small number of peptides, resulting in ‘holes’ in the TCR repertoire, which would facilitate evasion of T cell recognition by pathogens.

While there have been numerous studies delineating the structural footprint of the TCR on pMHC, there have only been a small number attempting to delineate the functionally more relevant energetic footprint of TCRs on pMHCs^10^−^16^, and their findings have been inconclusive. Some early studies were interpreted as suggesting that TCR contacts with MHC might contribute most of the binding energy^10^,^11^, whereas others suggested that TCR contacts with peptide are more important^12^. The approach used to determine the energetic footprint by all these studies was to remove the individual residue side chains by mutation to Ala or Gly and examine the effect of these mutations on the affinity of the TCR/pMHC interaction. Studies that mutated only TCR residues^10^,^11^,^13^,^14^ are difficult to interpret since many TCR residues make contact with both peptide and the MHC. This can be avoided by mutating MHC and peptide residues. However, these mutations can have effects on the adjacent MHC and/or peptide residues, complicating interpretation. One way to address this problem is to perform double-cycle mutagenesis^17^ where both TCR and pMHC residues are mutated and the effect of the individual and combined mutations on affinity measured^16^. While powerful, this approach has the disadvantage that it is only able to estimate the contributions of side chains to binding energy.

In the present study we attempted to measure the overall energetic contribution of TCR/MHC contacts to the TCR/pMHC interaction. We performed site-directed mutagenesis of HLA-A2 residues and measure the effect on the binding of 4 different HLA-A2-restricted TCRs. By comparing the binding of multiple TCRs to each mutant, we were able to rule out distal or conformational effects of mutations. We also compared single and double MHC mutations to confirm that the contributions to the binding energy were additive, as has been observed for other protein/protein interactions^18^. Conservative interpretation of these results enabled us to estimate a minimum energetic contribution of MHC residues to each the TCR/pMHC interaction. By examining the effect of MHC and peptide mutations on the kinetics of TCR binding, we provide evidence that both MHC and peptide residues are involved in the transition state complex and found no evidence for conserved MHC contacts, arguing against the proposed two-step binding mechanism of TCR engagement with pMHC.

## Results

**Figure 1:**
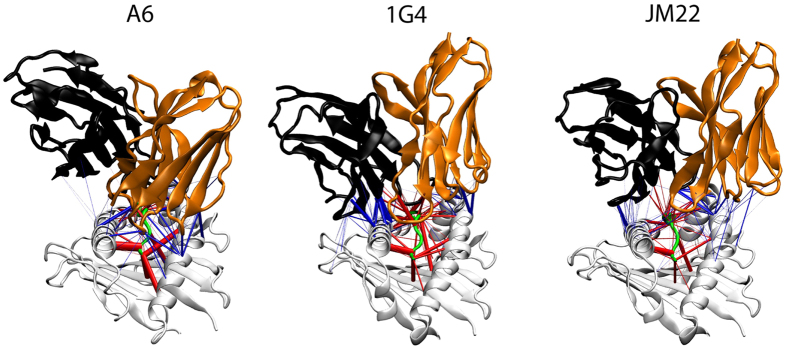
Predicted hydrogen bonds at the TCR/pMHC contact interface. The existence of hydrogen bonds between the indicated TCRs (Vα domain orange, Vβ domain black) and HLA-A2 (grey) presenting peptides (green) as predicted by molecular dynamics simulations (see Methods). Predicted hydrogen bonds involving TCR/MHC and TCR/peptide are represented by blue and red lines, respectively, with the line thickness proportional to the time the hydrogen bond was present. The HLA-A2 α1 and α2-helices are on the left and right of each structure, respectively.

We compared the contact interfaces of the A6^19^, 1G4^20^ and JM22^21^ TCRs with their cognate peptides presented by HLA-A2 (A*02:01) by performing multiple molecular dynamic simulations of each complex. This revealed a complex network of hydrogen (H) bonds that was unique for each TCR (Fig. 1). As expected, the significant predicted H bonds between the MHC and the TCRs (thicker blue lines) involved the α-helices flanking the peptide. For the A6 TCR the most prevalent H-bond interactions with the MHC involved R65 in the α1-helix and Q155 in the α2-helix. In contrast, for the 1G4 TCR the main H-bond interactions were with H70 and H73, both in the α1-helix. Finally, for the JM22 TCR the major H-bond interactions involved Q72 (α1) and Q155 (α2).

In order to investigate the contribution of individual HLA-A2 side chains to the binding energy, residues that could contribute to TCR binding were mutated to alanine (A) or, if they were already alanine, to glycine (G), effectively removing the corresponding side chains. To ensure that we included residues that might be involved in longer-range electrostatic interactions we selected HLA-A2 residues predicted to lie within 6 Å of the TCRs, based on published TCR-pMHC complex structures for the 1G4, A6 and JM22 TCRs (Tables S1–4). This included but was not limited to side chains predicted to form significant H-bonds with the TCRs in the molecular dynamics simulations (Fig. 1). Since no TCR-pMHC structure was available for the G10 TCR^22^ we analysed all the HLA-A2 mutants for effects on G10 binding.

**Figure 2:**
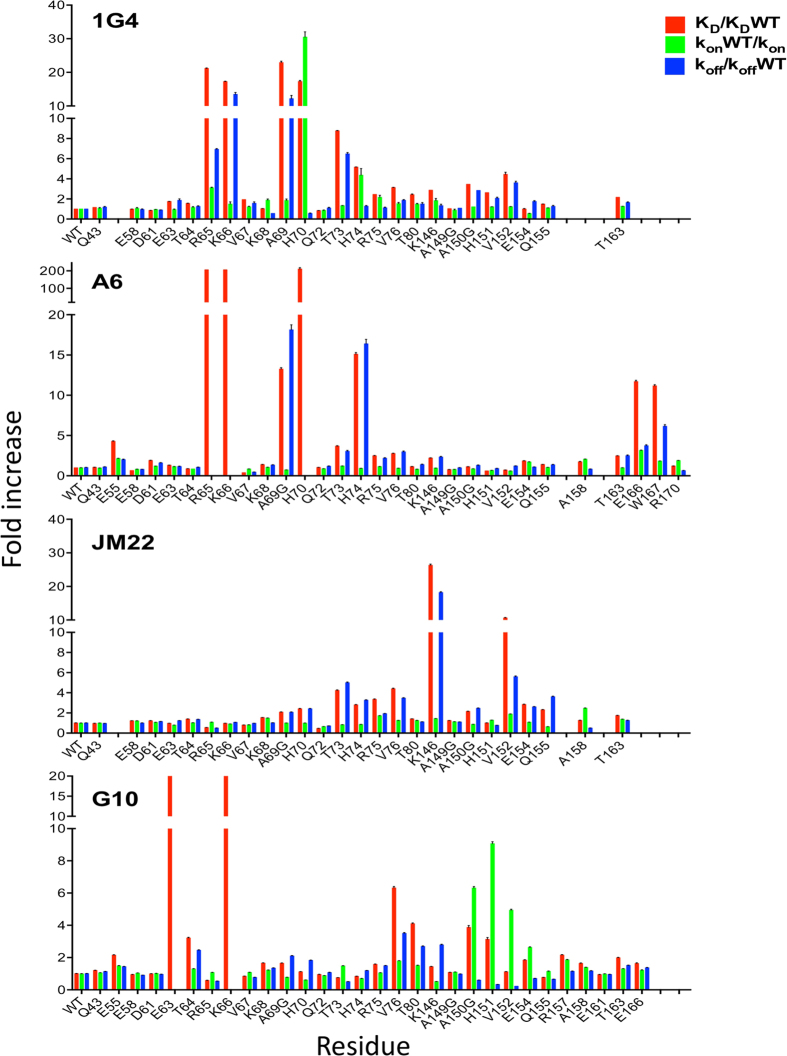
Binding properties of 1G4, A6, JM22 and G10 TCRs to the indicated HLA-A2 mutants. The K_D_, k_on_, and k_off_ of TCRs binding to the indicated HLA-A2 mutants, relative to binding to WT HLA-A2. These data are from Tables S1–4. The error bars represent range or SD from at least two independent SPR measurements.

Mutant and wild type (WT) pMHC were expressed as soluble proteins and the binding of the relevant TCRs was analysed by surface plasmon resonance (SPR). The dissociation constants (K_D_) and dissociation rate constants (k_off_) were determined directly (Figure S1) and the other parameters calculated from these (Tables S1 to 4). The effects of the mutation on binding parameters, normalized to WT pMHC, are plotted in Fig. 2. Values are expressed relative to the affinity and kinetics of TCR binding to WT pMHCs, which were consistent with values previously reported^19^,^20^,^21^,^22^,^23^.

### Different TCRs have very distinctive energetic footprints on HLA-A2

**Figure 3:**
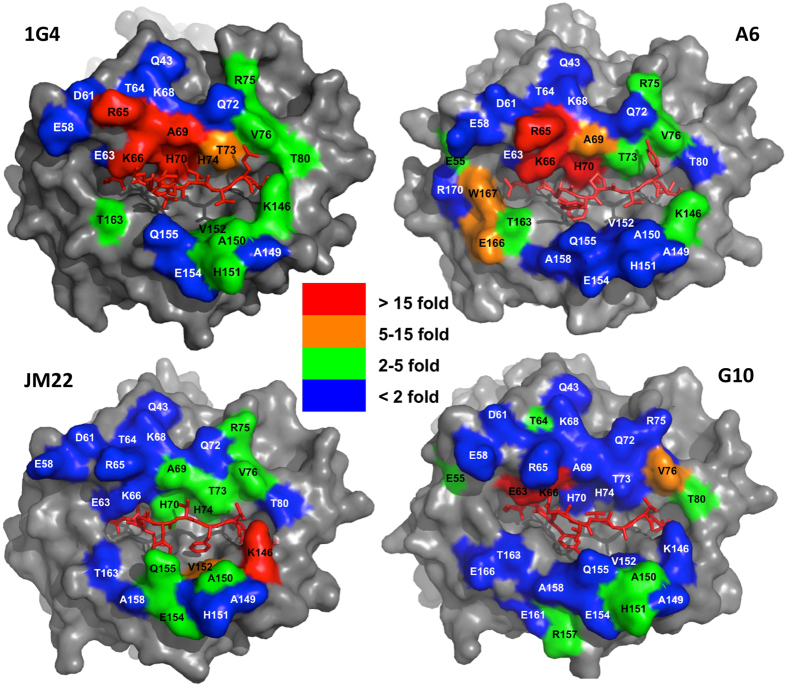
Energetic footprint of HLA-A2 restricted TCRs. Energetic footprints of the indicated TCRs on HLA-A2 presenting the appropriate cognate peptide. The residues are coloured according to the indicated fold-increase in K_D_ for the TCR binding the mutant compared with wild-type. HLA-A2 is shown with solid grey or coloured surfaces and the peptides as red stick models.

All the mutants were readily expressed and refolded, and none of the individual HLA-A2 mutations analysed affected binding to all four TCRs (Figs 2 and 3). This is strong evidence that the mutations did not have long-range effects or disrupt the overall HLA-A2 structure. For any given TCR only a subset of HLA-A2 mutations within the binding site reduced the binding energy, revealing the existence of an energetic footprint within the TCR binding footprint. For the 1G4 and A6 TCRs the energetic footprints lay in the α1 helix and involved residues R65, K66, A69 and H70. The energetic footprints were distinct, however, in that residues E166 and W167 also make substantial energetic contributions to the A6 binding whereas A150 and H151 make significant contributions to 1G4 binding. The energetic footprints of the JM22 and G10 TCRs are very different from the 1G4 and A6 footprints, and also differ from each other. Interestingly, while the R65A mutation decreased the affinity of the 1G4 and A6 TCRs, it increased the affinity of the JM22 and G10 TCRs (Tables S3 and S4). In summary, these results show that the energetic footprints for 4 TCRs restricted to the same MHC molecule can be very diverse.

### Energetic contributions between independent residues are additive

In principle, if all contact residues contribute independently to binding, and if it assumed that mutation to Ala/Gly only eliminates the energetic contribution of the mutated residue side chain, the sum of all the changes in binding energy (∑∆∆G) produced by each mutant should equal overall binding energy contributed by all contact residue side chains. While this additivity of has been confirmed for many protein/protein interactions^18^, it has not been been investigated for TCR/pMHC interactions.

**Figure 4:**
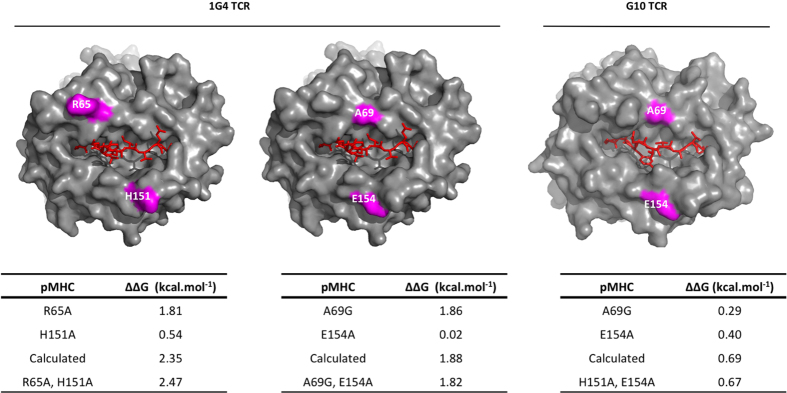
MHC residues make additive contributions to the binding energy. The indicated residues (purple) of HLA-A2 were mutated individually or in combination and their effect on the binding energy, relative to WT HLA-A2 (ΔΔG), determined by SPR. Calculated values for the double mutants are the sum of ΔΔG values measured for the single mutant. Data for single mutants is from Tables S1–S4. Data for double mutants represents the mean of at least three determinations with a SD less that 0.06 kcal.mol^−1^.

To test additivity, 2 pairs of non-adjacent amino acid residues were mutated and the affect on binding energy compaired with the single mutations (Fig. 4). The effects of the mutation of residue pairs on the binding energy were equal to the sum of the effect of the individual mutations. This supports the notion that, as observed for other protein/protein interactions^18^, the effects of individual mutations of non-adjacent MHC residues on TCR binding energy are additive.

### Estimating the minimum contribution of MHC residues to the overall binding energy

In principle, the additivity of the contribution of individual MHC residues to the binding energy makes it possible to estimate the overall contribution of MHC residues to the TCR/pMHC binding energy. However, there are two pitfalls with this approach that need to be avoided. The first is that many HLA-A2 mutants that reduced the TCR binding energy are of residues that make contact with the cognate peptide (Fig. 3 and Tables S1–4). It is therefore likely that the mutation is disrupting energetically important contacts between the TCR and the peptide. To avoid this pitfall we excluded these residues from our analysis. Because these excluded residues may contribute to the binding energy through their TCR contacts our calculation of the overall contribution of the MHC should be considered a minimum estimate.

A second pitfall is that, while it is reasonable to assume that mutations on non-adjacent contact residues are additive, it is more likely that mutations of adjacent contact residues, in a cluster, are not additive, since mutation of one residue is likely to influence the conformation of neighboring residues. This would complicate estimating their overall contribution to binding energy. This problem can be overcome by simultaneously mutating all residues in the cluster and measuring the change in binding energy, thereby estimating the contribution of the whole cluster.

**Figure 5:**
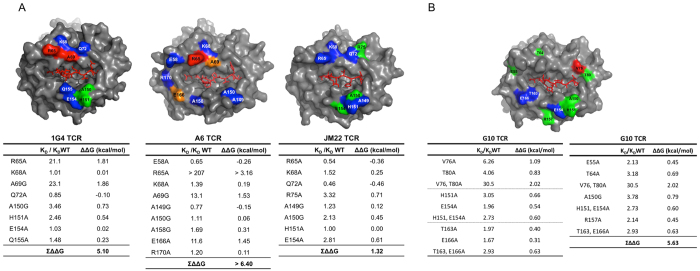
Estimating the total energetic contributions of TCR-contacting MHC residues. (**A**) 1G4, A6 and JM22-contacting residues on HLA-A2 that were included in the calculation are shown and the effects of their mutation tabulated below each figure. (**B**) For the G10 TCR, HLA-A2 residues that were selected for energetics calculation are shown. Residues in clusters were mutated together and the effect of the double mutation on binding energy determined (left table) and used to calculate the total contribution of HLA-A2 residues to binding energy (right table). The mutants are coloured using the same scheme as Fig. 1 according to fold effect on the mutation on the K_D_. Data on single mutants is from Tables S1–S4. Data on double mutants represents the mean of at least three determinations with a standard deviation of ΔΔG less that 0.06 kcal.mol^−1^. The errors for ∑ΔΔG were ≤0.18 kcal/mol.

When we looked for such clusters (residues with adjacent side chains in Fig. 3 that, when mutated, reduce the binding energy), taking care to exclude (for reasons outlined above) residues that make contact with the peptide (Tables S1–4), we found that there were no such clusters within the energetic footprints of the 1G4, JM22 and A6 TCRs (Fig. 5A).

In contrast, three relevant clusters were identified in the energetic footprint of G10 TCR: V76,T80; H151,E154; and T163,E166 (Fig. 5B). In order to estimate their combined contribution to binding energy both residues in these clusters were mutated simultaneously to Ala, and the effect on G10 binding energy determined (Fig. 5B). Interestingly, in the case of V76,T80 and T163, E166 clusters, the effects were additive whereas they were not with the H151,E154 cluster.

Having established that the effect on binding energy of mutation of non-adjacent residues are additive, and estimated the contribution of residue clusters to the binding energy, we next calculated the total energetic contribution of MHC residue side chains to TCR/pMHC interaction by adding contributions of individual residues and clusters. The residues and clusters which were included in the calculation are shown in Fig. 5. Since ΔΔG is the change in binding energy upon elimination by mutation, their contribution to the binding energy is −ΔΔG. We were unable to determine the precise ΔΔG for the R65A mutant on A6 binding but we estimated it to be greater than 3.16 kcal/mol. This is in agreement with a recent study by Piepenbrink *et al*. who reported that R65 contributed ~−5.6 kcal/mol to A6 TCR binding^16^.

The sum of all these contributions (ΣΔΔG) for each TCR represents an estimate of the contribution MHC side chain contacts to binding energy of the TCR/pMHC interaction. These estimates should be considered to be the lower limits or minimum contributions of MHC contacts, for two reasons. Firstly, because we excluded from the analysis all MHC residues that contact the peptide, and in some cases these residues also make direct contact with the TCR and are likely to contribute to the binding energy. Secondly, because, as noted above, we could only estimate the lower limit of the contribution of R65 to the binding of the A6 TCR.

For three of the TCR (1G4, A6, and G10) the minimum contributions of MHC residues to the TCR/pMHC binding energy are quite similar (−5.1, −6.4, and −5.63 kcal.mol^−1^, respectively). In contrast, our estimate for the minimum contribution of MHC residues to JM22 TCR binding is significantly lower at −1.3 kcal.mol^−1^. This suggests that the contribution of MHC residues in contact with the TCR to the binding energy can vary substantially.

### Estimating the relative contribution of MHC contacts to the total interaction energy

In order to evaluate the relative contribution of MHC versus peptide contacts it is necessary to obtain an estimate for the total interaction energy contributed by all contacts within the binding interface. Importantly, this is not the same as ΔG°, since ΔG° represents the sum of the receptor/ligand interaction energy and an unfavourable ‘association’ entropy arising from the reduction in volume of the reactants upon binding^24^,^25^. For ΔG°, where the reactants are assumed to be at the standard state concentration of 1 M, this has been estimated to be 4–6 kcal.mol^−1^ 16,24,25. Since this ‘association’ entropy value is unlikely to vary substantially between protein/protein complexes, we used it to estimate the total interaction energy from the ΔG° (Table 1). Based on these values, TCR/MHC contacts were calculated to contribute at least 11% of the interaction energy in the JM22/FLU-HLA-A2 complex and at least 41–48% of the total interaction energy in the three other TCR/pMHC interactions (Table 1).

**Figure 6:**
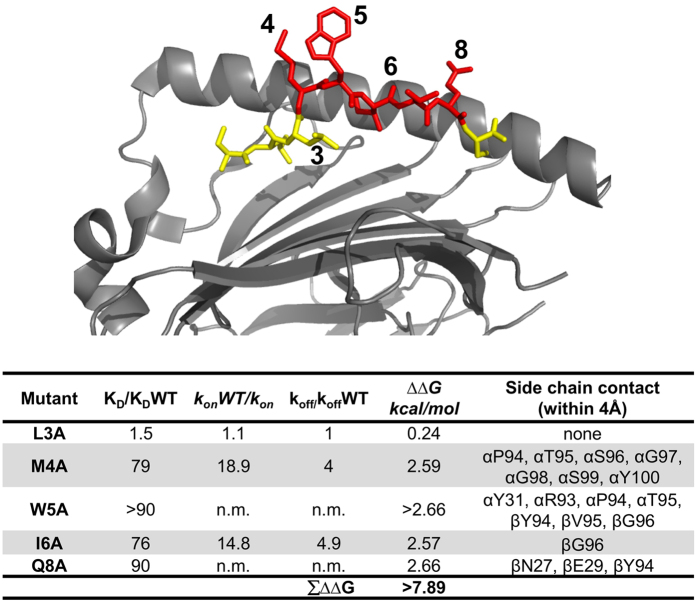
Effect of NEYSO-9V peptide mutations on the binding of 1G4 TCR to HLA-A2/NYESO-9V. Side-on view of HLA-A2 in complex with the NYESO peptide with the α2-helix omitted for clarity. Peptide residues in contact with 1G4 TCR are coloured red. Individual peptide residues were mutated to alanine and refolded with WT HLA-A2 heavy chains. The effects of the mutation on affinity and kinetics was determined by SPR and expressed relative to WT as in Table S1. To estimate the total contribution of side chains to binding energy (∑∆∆G) the combined contribution of the adjacent residues M4 and W5 was assumed to be >2.66 kcal/mol. ‘n.m.’ indicates not measureable.

As an alternative approach to estimating the total interaction energy we measured the contribution of peptide contacts to the binding energy by mutating 1G4 TCR contacting peptide side chains to Ala and measuring the change in binding energy (Fig. 6). We found that individual mutations of the NYESO-9V peptide, including mutations of neighbouring sidechains (M4A, W5A), resulted in very substantial changes in the 1G4 TCR binding energy; in some case (e.g. W5A) it was not possible to measure the affinity as it was too low. This made it difficult to assess the overall contribution of all peptide side-chains to the binding energy. However, if it is conservatively assumed that M4 and W5 together contributed at least −2.66 kcal.mol^−1^ (i.e. less than −2.66 kcal.mol^−1^), the total contribution of TCR-contacting peptide side chains to the binding energy can be calculated to be at least −7.89 kcal.mol^−1^ (Fig. 6). Combining the latter figure with the estimated contribution of the TCR-contacting HLA-A2 sidechains (Fig. 5) suggests that the total interaction energy for 1G4 TCR binding NYESO-9V-HLA-A2 is at least −13 kcal.mol^−1^ (Table 1). This agrees well with calculated total interaction energy of 12.5 ± 1.12, Table 1), validating the latter approach for estimating the total TCR/pMHC interaction energy. The relative contribution of MHC contacts to the total interaction energy for the 1G4 TCR/NYESO-9V-HLA-A2 complex was calculated to be at least 39%, which agrees well with the estimate (at least 41%) made using the calculate total interaction energy (Table 1).

### Contribution of MHC residues to the transition state complex

According to transition state theory the association rate constant (k_on_) is determined by the energy required to form a transition state complex of the interacting molecules. Examining the effect of mutations to Ala or Gly of contact residues on the k_on_ can therefore be used to probe the contribution of these residues to the transition state complex, delineating a kinetic ‘footprint’. Such an analysis of a mouse TCR/pMHC interaction indicated that mutations of MHC residues often decrease the k_on_, which was interpreted to support a ‘two-step’ binding model whereby the TCR inititally contacts MHC residues (in the transition state complex) and subsequently engages peptide in the final complex^12^.

**Figure 7:**
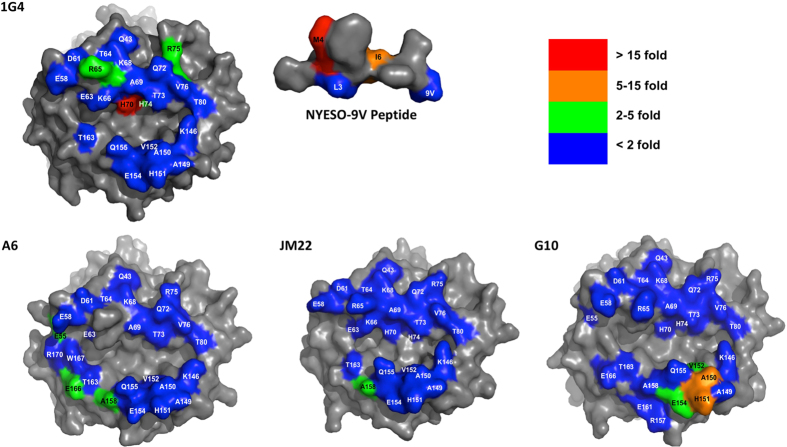
Kinetic footprints of HLA-A2 restricted TCRs. Kinetic footprints of the indicated TCRs (1G4, A6, JM22 and G10). The residues on HLA-A2 that were mutated are coloured according to the indicated fold decrease in k_on_ of the mutant compared with wild-type. The NYESO-9V peptide residues are similarly coloured. Residues not mutated, or mutation of which was uninformative, are in gray.

We compared the effect of mutation on HLA-A2 residues on the k_on_ for the four TCR/pMHC interactions in this study (Tables S1–4 and Figs 2 and 7). Mutation of only a small proportion of MHC residues decreased the k_on_ more than 2 fold, and these residues were different for the different TCRs (Figs 2 and 7). Furthermore, some of the mutants that decreased the k_on_ were of residues (H70A, H74A, V152A) that contacted the peptide but not the TCR (Tables S1–4), indicating that they are unlikely to contribute to TCR contacts in the transition state complex; more likely they affect the k_on_ by altering the conformation of the peptide. Some of the mutations which decreased the k_on_ were of residues that were within 6 Å of the TCR but did not make direct contacts. These residues were often charged (E55, R75) or adjacent to charged residues (A158), suggesting that these mutation were perturbing long-rang electrostatic interactions, which can have a major impact on the k_on_^26^. Interestingly, mutations of two residues of the NYESO-9V peptide that make TCR contact did substantially decrease the k_on_ for 1G4 binding (M4 and I6 in Fig. 6), suggesting that their sidechains form TCR contacts in the transition state.

Taken together, the effect of mutations of MHC and peptide residues on TCR k_on_ argue against a dominant role for TCR/MHC contacts in the transition state complex, as previously proposed^12^, and suggest instead that TCR/peptide contacts can also contribute.

## Discussion

The primary focus of this study was to characterise the energetic footprint of TCRs on pMHC and use this to estimate the relative contribution of TCR interactions with the MHC versus peptide to the binding energy of TCR/pMHC interactions. While there have been numerous studies delineating the structural details of the TCR interaction with pMHC, also termed the structural footprint, only a handful of studies have attempted the much more challenging task of defining the energetic footprint. These involved performing alanine/glycine mutagenesis of the TCR^10^,^11^,^13^,^27^, the peptide-MHC residues^12^,^28^,^29^ or a combination of the two^15^,^16^. The principle underlying this type of analysis is that mutation to alanine or glycine, by removing most or all the residue side-chain, can reveal the contribution of that side chain to binding. A drawback of mutating TCR residues is that many TCR residues contact both the peptide and MHC, making it impossible to distinguish their contributions. For this reason the focus of this study was on examining the effect of MHC (and peptide) mutants on TCR binding.

This study compared the contribution of HLA-A2 class I residues to the binding of 4 different TCRs. We found that there is considerable variation in the energetic footprints of the 4 different TCRs studied on HLA-A2. There were no residues or groups of residues that contributed to the binding energy for all 4 TCRs. This is consistent with the observation from numerous structural studies that, while there may be conserved contacts between TCRs containing particular V segments and particular MHC alleles^5^,^30^, there are no generally-conserved contacts^8^. The recent observation that TCRs can bind in the opposite orientation supports this^31^.

MHC mutations may affect the TCR-pMHC binding by disrupting MHC contacts with TCR and/or affecting the peptide antigen structure and thus indirectly disrupting peptide-TCR contacts. To determine the energetic contribution of MHC alone, only residues making exclusive contact with TCR were considered. This approach allows an estimation of the MHC’s contribution to TCR-pMHC interaction independent of the peptide’s involvement. It is, however, a conservative estimate, and should be considered a lower limit of the true contribution.

In principle, mutation to alanine or glycine can reduce the binding energy by several mechanisms^32^. These include local effects such as loss of favorable contacts and distal effects such as changes in conformational flexibility. They include alterations in the stability of the bound complex or of the unbound pMHC. In our analysis we have assumed that the primary effect of the mutations is to locally peturb favorable interactions. While this assumption is supported by the fact that the HLA-A2 mutations did not reduce protein expression, and only perturbed binding of some of the TCRs, we cannot rule out other possibilities.

To estimate the total contribution of MHC residues to the TCR-pMHC affinity we took advantage of the fact that within a protein/protein interface changes in binding energy observed following point mutation of non-adjacent residues to Alanine are typically additive^18^. We confirmed that this additivity also applied to the TCR/pMHC complex by demonstrating that changes in free energies of binding following simultaneous mutation of two residues not in contact was equal to the sum of change in binding energies observed when they were mutated individually. One caveat is that we cannot rule out the possibility that mutations of HLA-A2 are not all additive because of long range effects on the conformation or dynamics of the HLA-A2 or the interacting TCR. One way of overcoming this difficulty is double-cycle mutagenesis, where both TCR and pMHC residues are mutated and the effect of single (TCR only or pMHC only) and double mutants (TCR and pMHC) are compared. This approach makes it possible to deduce the interaction energy between two side chains independently of effects on other residues. These studies are technically challenging, however because the introduction of two mutations frequently decreases the affinity so much that accurate measurements become impossible. The only double cycle mutagenesis of a TCR/pMHC interaction that has been performed to date is that Piepenbrook *et al*. (2013) who studied the A6 and B7 TCRs binding to Tax-HLA-A2^16^. They took advantage of the existence of high affinity variants of these TCRs, which enabled them to measure the combined effect of two mutations.

Mutational effects of adjacent or ‘clustered’ residues may not be additive as they can influence each other, and this needs to be taken into account when calculating their contribution to the overall binding energy. No relevant clusters were identified in the case of 1G4, A6 and JM22 binding but three clusters of two residues each were identified in the G10 energetic footprint. Their contribution to G10 binding energy was determined by simultaneously mutating both residues in each cluster and measuring the resulting change in G10 binding energy.

Using this approach we proceeded to calculate the overall contribution of MHC residues to the binding of the 4 different TCRs. Our results suggest that the relative energetic contribution of MHC to TCR binding can vary substantially, ranging from as little a −1.3 kcal.mol^−1^ to at least −6.4 kcal.mol^−1^. For three of the TCRs the MHC contribution was quite similar (−5.4 to −6.4 kcal.mol^−1^) whereas it was much lower for the JM22 TCR (−1.3 kcal.mol^−1^).

Estimating the relative contribution of MHC contacts to the total interaction energy required an estimation of the latter. As noted in the Results, it is not appropriate to express the contribution of MHC contacts as a proportion of the ΔG° since the latter includes an arbitrary ‘association’ entropy, the value of which is determined by the assumed concentration of the reactants^24^,^25^. Using an estimate of the latter we calculated the total interaction energy from the ΔG°. This approach is supported by the fact that the value calculated for the 1G4/NYESO-9V-HLA-A2 interaction agreed well with the value determined from the mutagenesis data (Table 1).

We found that HLA-A2 contacts contributed from 40–48% of the binding energy for the 1G4, A6 and G10 TCRs, but a little as 11% of the binding energy for the JM22 TCR. This is lower the percentage contribution of MHC to the binding interface, which ranges from 62 to 72% for these TCRs^8^. While previous studies have not attempted to quantitate the overall contribution of MHC contacts to the binding energy some comparisons can be made. Site-directed mutagenesis studies of the murine 2C TCR binding to allogeneic^10^ and syngeneic^11^ ligands were interpreted as indicating that the MHC contacts provided the bulk of the binding energy; however these studies relied on indirect measurements of binding energy. A more quantitative analysis of the murine 2B4 TCR binding cognate pMHC was interpreted as showing that peptide contacts were dominant^12^. A more recent analysis of the A6 TCR/Tax-HLA-A2 interaction by double cycle mutagenesis indicated that interactions between TCR and MHC side chains contribute the bulk (~70%) of the binding energy^16^. One caveat is that there are several contacts between the A6 TCR backbone and the peptide that the method does not measure.

The low contribution of MHC contacts to JM22 TCR binding was somewhat unexpected given that the peptide is somewhat ‘featureless’ with small side chains such as Gly, Val, Thr exposed to the TCR^21^. The results are, however, consistent with molecular dynamics simulations which revealed numerous hydrogen bonds between the TCR and peptide and relatively few/weak bonds between the TCR and MHC (Fig. 1). One potential explanation is that JM22 TCR contacts with MHC backbone residues, which were not assessed in this study, are particularly important contributors to the binding energy. Interestingly the JM22 TCR makes proportionally more contacts with MHC backbone atoms than either 1G4 or A6 (Table S5).

The two-step model of TCR binding was based on reports that mutations of MHC residues have substantial effects on the k_on,_ indicating that TCR/MHC contacts form in the transition state complex^12^. In contrast, we found that relatively few MHC mutations affected the k_on_ while the k_on_ of the 1G4 TCR was greatly reduced by mutations of the cognate peptide. These results, and similar results reported by Baker *et al*.^4^, suggest that formation of TCR/MHC contacts in the transition state complex is not a general feature of TCR/pMHC interactions, and that TCRs may also form contacts with the peptide in the transition state. Thus the two-step binding model is not a general feature of TCR binding.

In conclusion, we report here that the energetic footprint of 4 TCRs on HLA-A2 is highly variable, and estimate that HLA-A2 contacts can contribute less than half of the total interaction energy. We also find that the TCR/HLA-A2 contacts do not seem to make a major contribution the transition state complex whereas TCR contacts with the peptide can form during the transition state. Our results, together with previous reports, suggest that, as is the case with the structural basis of TCR/pMHC recognition, there are few general rules governing the energetics of TCR/pMHC binding.

## Methods

### Molecular dynamics simulations and structural visualization

The experimentally determined X-ray structure of A6/LLFGYPVYV/HLA-A*02:01 (accession code *1AO7*), JM22/GILGFVFTL/HLA-A*02:01 (accession code *1OGA*), and 1G4/SLLMWITQC/HLA-A*02:01 (accession code *2BNR*) were extracted from the Protein Data Bank (PDB)^34^. Molecular Dynamics simulations were carried out using Gromacs 4^35^ and the GROMOS 53a6 force-field^36^: all three complexes were immersed into separate dodecahedronic explicit SPC water^37^ boxes. The box size was chosen to allow for a minimum distance of 1.2 nm between protein complex and box boundary. Randomly choosen water molecules were replaced with Na and Cl ions to achieve a salt concentration of 0.15 mol/liter and a neutral charge. The systems were energetically minimised using the steepest descent method and subsequently warmed up to 310 K using position restraints. Then production runs were carried out for a real time of 100 ns per complex. Ten replicas (identical simulations but different seeds) per complex were performed yielding a total simulation time of 3 000 ns.

H-bond visualisations (Fig. 1) were created using H-Vis-3D (Knapp B, Zhang H, van der Merwe PA and Deane CM, in preparation). Figures 2, 4, 5, 6 and 7 were drawn using the PyMOL Molecular Graphics System (Schrödinger, LLC). Contacts (Tables S1–5) were identified with Swiss-pdb viewer (Swiss Institute of Bioinformatics) or PyMOL, using a distance threshold of 4 Å.

### TCR, MHC and peptide constructs

The four TCRs used are: 1G4, A6, JM22 and G10^19^,^20^,^21^,^22^. The constructs were obtained from Vincenzo Cerundolo, Yvonne Jones and Guillaume Stewart-Jones laboratories (1G4, JM22 and G10) (University of Oxford, UK) and Brian Baker’s laboratory (A6) (University of Notre Dame, USA). Their sequences and structures can be found in the following PDB files: 2BNR (1G4), 1AO7 (A6), 1OGA (JM22). The 1G4, A6 and JM22 TCRs recognize peptides derived from the NY-ESO-1 tumour antigen, the HIV and the influenza matrix protein (MP), respectively. The G10 TCR is specific for an HIV GAG p17 peptide SLFNTVATL, and utilizes the TRAV20 (Vα) and TRB5-1 (Vβ) gene segments^22^. The structure of the G10 TCR ligand HLA-A2: SLFNTVATL can be found in the PDB file 2V2W.

The peptides purchased from GenScript (GenScript Co. USA) were synthesised by standard solid-phase chemistry on a multiple peptide synthesiser using F-moc for transient NH2-terminal protection. Minimal peptide was synthesized by ALTA Bioscience, Birmingham, UK. All peptides were >90% pure, as indicated by analytical HPLC. Their sequences are:

NYESO-9V (for 1G4 TCR): SLLMWITQV

TAX (for A6 TCR): LLFGYPVYV

MP (for JM22 TCR): GILGFVFTL

GAG (for G10 TCR): SLFNTVATL

HLA-A2 (A*02:01) heavy chain (residues 1–278) with C-terminal BirA tag and β2-microglobulin were expressed as inclusion bodies in *E. coli*, refolded *in vitro* in the presence of one of the four synthesised peptides, and purified using size-exclusion chromatography^38^. Purified pMHC was biotinylated *in vitro* by BirA enzyme (Avidity). Amino-acid substitutions were introduced in HLA-A2 expressing plasmid using a QuickChange site directed mutagenesis kit (Stratagene). Modified HLA-A2 proteins were all refolded with the peptides. The 1G4 TCR subunits, α and β, were expressed in *E. coli* as inclusion bodies, refolded *in vitro*, and purified using size exclusion chromatography as previously described^39^.

### Surface plasmon resonance

Binding of each TCR to pMHC variants was analysed by SPR on a BIAcore 3000 (GE Healthcare), as previously described^23^. Unless otherwise stated all experiments were performed at 25 °C and using a flow rate of 10 μL/minute in HBS-EP buffer (0.01 M HEPES buffer (pH 7.4), 0.15 M NaCl, 0.005% Surfactant P20). Biotinylated pMHC was immobilised to CM5 sensor chip (GE Healthcare) indirectly by covalently coupled streptavidin at various levels (~250 RU for kinetics and ~1200 RU for affinity measuring). To determine affinity of each TCR for its ligands, equilibrium binding was measured for graded concentrations of TCR. The K_D_ value was obtained by non-linear curve fitting using Origin (OriginLab) to the Langmuir binding isotherm,


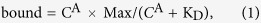


where “bound” is the equilibrium binding in RUs at injected TCR concentration C^A^ and Max is the maximum binding (RUs). The effect of MHC mutations on the binding energy (ΔΔG) was calculated from the relationship


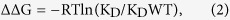


where R is the Gas constant (1.987 cal.mol^−1^.K^−1^) and T is the absolute temperature (K). At 25 °C RT is 0.592 kcal.mol^−1^.

Kinetics of TCR/pMHC interaction was measured by injecting TCR at a flow rate of 50 μl/minute over low levels of immobilised pMHC (250 RU) to minimise mass transport effects. Dissociation rate constant k_off_ was determined by curve fitting to the 1:1 Langmuir binding model using BIAevaluation (BIAcore software) (31). Association rate constant k_on_ was calculated using k_on_ = k_off_/K_D_.

All binding parameter determinations were performed at least twice. For clarity errors are not shown in Tables S1–S4 and Fig. 5. The range or standard deviation was less than 10% of mean values for K_D_ and k_off_ measurements, less than 14% for the calculated k_on_, and less than 0.06 kcal.mol^−1^ for ΔΔG measurements. Errors for ΣΔΔG determinations were calculated by standard error propagation methods.

## Additional Information

**How to cite this article**: Zhang, H. *et al*. The contribution of major histocompatibility complex contacts to the affinity and kinetics of T cell receptor binding. *Sci. Rep.* **6**, 35326; doi: 10.1038/srep35326 (2016).

## Supplementary Material

Supplementary Information

## Figures and Tables

**Table 1:** Estimating the relative contribution of TCR/MHC contacts to the total TCR/pMHC interaction energy.

	ΔG°^1^	Total_calc_^2^	Total_exp_^3^	MHC^4^	MHC (%)^5^
1G4/NYESO-9V-HLA-A2	−7.5 ± 0.12	−12.5 ± 1.12	−13 ± 0.21	−5.1 ± 0.17	41 ± 4, 39 ± 2
A6/TAX-HLA-A2	−8.26 ± 0.06	−13.26 ± 1.06	n.d.	−6.4 ± 0.18	48 ± 4
JM22/MP-HLA-A2	−7.2 ± 0.06	−12.2 ± 1.06	n.d.	−1.32 ± 0.17	11 ± 2
G10/GAG-HLA-A2	−6.93 ± 0.06	−11.93 ± 1.06	n.d.	−5.63 ± 0.16	47 ± 4

All data except the final column are in units of kcal.mol^−1^.

^1^The standard state binding energy (ΔG°) was calculated from the K_D_ using the relationship ΔG° = RTlnK_D_, where K_D_ is expressed in M.

^2^The total TCR/pMHC interaction energy (Total_calc_) was calculated from the relationship. Total_calc_ = ΔG°− association entropy. where the association entropy was assumed to 5 ± 1 kcal.mol^−1^ at the standard state, and to be the same for all TCR/pMHC interactions^16^,^24^,^25^.

^3^The experimentally determined total TCR/pMHC interaction energy (Total_exp_) for the 1G4 TCR was obtained by adding the contributions of the TCR/MHC and TCR/peptide determined in Figs 5A and 6, respectively. ‘n.d.’ not determined.

^4^The contribution of MHC side chain to the binding energy. Data from Fig. 5.

^5^The % contribution of MHC contacts to the total interaction energy. For the 1G4 TCR the two values shown for MHC (%) use Total_calc_ and Total_exp_, respectively.
