# Supplementary material for: The contribution of major histocompatibility complex contacts to the affinity and kinetics of T cell receptor binding
Source: Sci Rep. 2016 Oct 13;6:35326. doi: 10.1038/srep35326 (PMC5062128; doi:10.1038/srep35326)
Supplement: Supplementary Information [file srep35326-s1.doc]

**Supplementary Material**

**for**

**The contribution of major histocompatibility complex contacts to the affinity and kinetics of T cell receptor binding**

*Hao Zhang, Hong-Sheng Lim, Berhard Knapp, Charlotte M. Deane, Milos Aleksic, Omer Dushek, and P. Anton van der Merwe*

**Figure S1. Affinity and kinetic measurements**

(A) Representative equilibrium binding assay. Increasing concentrations of TCR were flowed over surfaces with cognate pMHC immobilized at ~1200 Response Units (RU) as well as control surfaces. The binding responses at each concentration were obtained by subtracting the responses in the reference flow cells and overlaid. (B) The binding at equilibrium (red circles) was plotted against increasing TCR concentrations and the Langmuir binding isotherm fitted to the data (red line) to obtain the K_D_. To demonstrate assay robustness the analysis was always repeated by injecting decreasing concentrations of TCR (black squares). (C) Representative kinetic assay. TCRs were flowed over surfaces with pMHC immobilized at ~250 RU, followed by a washing phase when only buffer is injected. The figure depicts the level remaining bound (black line) following the end of the injection (t=0) normalized to the amount bound at t = 0. The dissociation rate constant k_off_ was determined by fitting a monoexponential decay curve (red line) using BIAevaluation software.

**A**

**B**

**C**

| ***pMHC*** | ***TCR contacts*** | ***Peptide contacts*** | ***K_D_/K_D_WT*** | ***k_on_WT/k_on_*** | ***k_off_/k_off_WT*** | ***∆∆G***  ***kcal/mol*** |
| --- | --- | --- | --- | --- | --- | --- |
| Q43A |  |  | 1.15 | 1.01 | 1.14 | 0.08 |
|  |  |  |  |  |  |  |
| E58A |  |  | 1.00 | 1.01 | 0.99 | 0.00 |
| D61A |  |  | 0.86 | 0.95 | 0.91 | -0.09 |
| E63A |  | S1, L2 | 1.76 | 0.89 | 1.97 | 0.34 |
| T64A |  |  | 1.58 | 1.21 | 1.30 | 0.27 |
| R65A | α: G98, S99  β: Y47, V49, D55 |  | 21.1 | 3.06 | 6.89 | 1.81 |
| K66A | α: G98, Y100 | S1, L2, L3, M4 | 17.3 | 1.33 | 13.0 | 1.69 |
| V67A |  | L2 | 1.94 | 1.16 | 1.68 | 0.39 |
| K68A | β: I53 |  | 1.01 | 1.79 | 0.57 | 0.01 |
| A69G | α: Y100  β: V49 |  | 22.9 | 1.78 | 12.9 | 1.86 |
| H70A |  | L3 | 17.5 | 29.4 | 0.60 | 1.70 |
| Q72A | β: E29, V49, G50, A51, T70 |  | 0.85 | 0.82 | 1.04 | -0.10 |
| T73A | β: E29, V95 | I6, Q8 | 8.74 | 1.33 | 6.58 | 1.28 |
| H74A |  |  | 5.19 | 3.89 | 1.33 | 0.98 |
| R75A |  |  | 2.45 | 2.31 | 1.06 | 0.53 |
| V76A |  | Q8 | 3.11 | 1.62 | 1.92 | 0.67 |
| T80A |  | C9 | 2.47 | 1.54 | 1.61 | 0.54 |
| K146A |  | Q8, C9 | 2.87 | 1.99 | 1.44 | 0.63 |
| A149G |  |  | 1.03 | 0.96 | 1.08 | 0.02 |
| A150G | α: Q51  β: N97 |  | 3.46 | 1.21 | 2.85 | 0.73 |
| H151A | α: Q51, Q54 |  | 2.46 | 1.15 | 2.15 | 0.54 |
| V152A |  | T7 | 4.59 | 1.24 | 3.72 | 0.90 |
| E154A | α: S53 |  | 1.03 | 0.57 | 1.81 | 0.02 |
| Q155A | α: Y31, T95 |  | 1.48 | 1.11 | 1.33 | 0.23 |
| T163A |  |  | 2.17 | 1.28 | 1.70 | 0.46 |

**Table S1. Effect of HLA-A2 mutations on the binding of 1G4 TCR to HLA-A2/ NYESO-9V**

Mutated residues on the HLA-A2 heavy chain and their corresponding contact residues on 1G4 TCR α and/or β subunits or NYESO peptide are listed. The binding properties shown were averaged from at least two independent experiments and normalized to the WT K_D_, k_on_ , and k_off_ values, which were 3.2 μM, 2.4 x 10^4^ M^-1^.s^-1^ and 0.10 s^-1^, respectively.

| ***pMHC*** | ***TCR contacts*** | ***Peptide contacts*** | ***K_D_/K_D_WT*** | ***k_on_WT/k_on_*** | ***k_off_/k_off_WT*** | ***∆∆G***  ***kcal/mol*** |
| --- | --- | --- | --- | --- | --- | --- |
| Q43A |  |  | 1.05 | 0.98 | 1.07 | 0.03 |
|  |  |  |  |  |  |  |
| E55A |  |  | 4.24 | 2.16 | 1.96 | 0.86 |
| E58A | α: K1 |  | 0.65 | 0.83 | 0.78 | -0.26 |
| D61A |  |  | 1.90 | 1.22 | 1.56 | 0.38 |
| E63A |  | L1, L2 | 1.31 | 1.15 | 1.13 | 0.16 |
| T64A |  |  | 0.88 | 0.85 | 1.03 | -0.08 |
| R65A | α: T97, D98, W101, G102 |  | >206 | n/m | n/m | >3.16 |
| K66A | α: Q30, D99 | L1, L2, F3 | >206 | n/m | n/m | >3.16 |
| V67A |  | L2 | 0.39 | 0.85 | 0.45 | -0.56 |
| K68A | α: W101 |  | 1.39 | 1.07 | 1.30 | 0.19 |
| A69G | α: W101 |  | 13.1 | 0.74 | 17.69 | 1.53 |
| H70A |  | F3 | 206 | n/m | n/m | 3.16 |
| Q72A |  | Y8 | 1.03 | 0.89 | 1.16 | 0.02 |
| T73A |  | P6, V7, Y8 | 3.65 | 1.22 | 2.98 | 0.77 |
| H74A |  |  | 15.0 | 0.94 | 15.99 | 1.60 |
| R75A |  |  | 2.47 | 1.16 | 2.13 | 0.53 |
| V76A |  | Y8 | 2.74 | 0.94 | 2.90 | 0.60 |
| T80A |  | V9 | 1.14 | 0.83 | 1.37 | 0.08 |
| K146A |  |  | 2.18 | 0.96 | 2.27 | 0.46 |
| A149G | β: R102 |  | 0.77 | 0.80 | 0.97 | -0.15 |
| A150G | β: G101, R102 |  | 1.11 | 0.88 | 1.27 | 0.06 |
| H151A |  |  | 0.60 | 0.68 | 0.88 | -0.30 |
| V152A |  | V7 | 0.72 | 0.61 | 1.18 | -0.20 |
| E154A |  |  | 1.84 | 1.74 | 1.06 | 0.36 |
| Q155A | β: G101, P103 | F3, Y5 | 1.40 | 1.05 | 1.34 | 0.20 |
| A158G | α: Y50 |  | 1.69 | 2.08 | 0.81 | 0.31 |
| T163A | α: Q30, K68 | L1 | 2.44 | 1.00 | 2.44 | 0.53 |
| E166A | α: N52, K68 |  | 11.6 | 3.18 | 3.65 | 1.45 |
| W167A | α: R27 | L1 | 11.1 | 1.85 | 5.99 | 1.42 |
| R170A | α:R26 |  | 1.20 | 1.91 | 0.63 | 0.11 |

**Table S2. Effect of HLA-A2 mutations on the binding of A6 TCR to HLA-A2/ TAX**

Mutated residues on the HLA-A2 heavy chain and their corresponding contact residues on A6 TCR α and/or β subunits or TAX peptide are listed. The binding properties shown were averaged from least two independent experiments and normalized to the WT K_D_, k_on_ , and k_off_ values, which were 0.88 μM, 1.2 x 10^5^ M^-1^.s^-1^, and 0.10 s^-1^, respectively.

| ***pMHC*** | ***TCR contacts*** | ***Peptide contacts*** | ***K_D_/K_D_WT*** | ***k_on_WT/k_on_*** | ***k_off_/k_off_WT*** | ***∆∆G***  ***kcal/mol*** |
| --- | --- | --- | --- | --- | --- | --- |
| Q43A |  |  | 0.96 | 1.01 | 0.95 | -0.03 |
|  |  |  |  |  |  |  |
| E58A |  |  | 1.21 | 1.22 | 0.99 | 0.11 |
| D61A |  |  | 1.22 | 1.07 | 1.14 | 0.12 |
| E63A |  | G1, I2 | 0.97 | 0.79 | 1.22 | -0.02 |
| T64A |  |  | 1.37 | 1.03 | 1.34 | 0.19 |
| R65A | β: Q58 |  | 0.54 | 1.09 | 0.50 | -0.36 |
| K66A |  | G1, I2, L3, G4 | 0.96 | 0.91 | 1.05 | -0.02 |
| V67A |  | I2 | 0.79 | 0.82 | 0.96 | -0.14 |
| K68A | β: D56 |  | 1.52 | 1.50 | 1.01 | 0.25 |
| A69G | β: D56 | V6 | 2.06 | 1.00 | 2.05 | 0.43 |
| H70A |  | I2, L3, V6 | 2.39 | 1.00 | 2.39 | 0.52 |
| Q72A | β: I53, V54, N55 |  | 0.46 | 0.65 | 0.71 | -0.46 |
| T73A | β: I53 | V6, F7, T8 | 4.19 | 0.85 | 4.95 | 0.85 |
| H74A |  |  | 2.78 | 0.86 | 3.23 | 0.60 |
| R75A | β: N55 |  | 3.32 | 1.74 | 1.91 | 0.71 |
| V76A | β: I53, V54 | T8 | 4.35 | 1.27 | 3.43 | 0.87 |
| T80A |  | L9 | 1.39 | 1.26 | 1.10 | 0.20 |
| K146A |  | T8, L9 | 26.0 | 1.44 | 18.0 | 1.93 |
| A149G | β: Y101 |  | 1.23 | 1.13 | 1.09 | 0.12 |
| A150G | β: R98, Y101 |  | 2.13 | 0.87 | 2.44 | 0.45 |
| H151A | α: V51  β: R98, Y101 |  | 1.00 | 1.29 | 0.77 | 0.00 |
| V152A | β: R98 | F7 | 10.5 | 1.90 | 5.54 | 1.39 |
| E154A | α: S31, V51 |  | 2.81 | 1.09 | 2.57 | 0.61 |
| Q155A | α: S31, G94  β: R98, S100 | F5 | 2.28 | 0.64 | 3.57 | 0.49 |
| A158A |  |  | 1.25 | 2.49 | 0.50 | 0.13 |
| T163A |  |  | 1.72 | 1.38 | 1.25 | 0.32 |

**Table S3. Effect of HLA-A2 mutations on the binding of JM22 TCR to HLA-A2/ MP**

Mutated residues on the HLA-A2 heavy chain and their corresponding contact residues on JM22 TCR α and/or β subunits or MP peptide are listed. The binding properties shown were averaged from at least two independent experiments and normalized to the WT K_D_, k_on_ , and k_off_ values, which were 5.3 μM, 2.8 x 10^4^ M^-1^.s^-1^ and 0.15 s^-1^, respectively.

| ***pMHC*** | ***Peptide Contact*** | ***K_D_/K_D_WT*** | ***k_on_WT/k_on_*** | ***k_off_/k_off_WT*** | ***∆∆G***  ***kcal/mol*** |
| --- | --- | --- | --- | --- | --- |
| Q43A | None | 1.20 | 1.06 | 1.12 | 0.11 |
| **E55A*** | **None** | **2.13** | **1.50** | **1.42** | **0.45** |
| E58A | None | 0.94 | 1.05 | 0.90 | -0.04 |
| D61A | None | 0.98 | 1.03 | 0.96 | -0.01 |
| E63A | S1, L2 | >21.9 | n/m | n/m | >1.83 |
| **T64A*** | **None** | **3.18** | **1.31** | **2.43** | **0.69** |
| R65A | S1, L2 | 0.58 | 1.08 | 0.54 | -0.32 |
| K66A | S1, L2, N4 | >21.9 | n/m | n/m | >1.83 |
| V67A | None | 0.84 | 1.09 | 0.77 | -0.11 |
| K68A | None | 1.64 | 1.23 | 1.33 | 0.29 |
| A69G | None | 1.63 | 0.79 | 2.08 | 0.29 |
| H70A | Y3, V6 | 1.11 | 0.61 | 1.81 | 0.06 |
| Q72A | None | 0.95 | 0.89 | 1.07 | -0.03 |
| T73A | V6, T8 | 0.75 | 1.50 | 0.50 | -0.17 |
| H74A | None | 0.83 | 0.71 | 1.18 | -0.11 |
| R75A | None | 1.57 | 1.06 | 1.48 | 0.27 |
| **V76A*** | **None** | **6.26** | **1.81** | **3.47** | **1.09** |
| **T80A*** | **None** | **4.06** | **1.52** | **2.66** | **0.83** |
| K146A | T8, 9L | 1.42 | 0.52 | 2.76 | 0.21 |
| A149G | None | 1.07 | 1.11 | 0.97 | 0.04 |
| **A150G*** | **None** | **3.78** | **6.37** | **0.59** | **0.79** |
| **H151A*** | **None** | **3.05** | **9.16** | **0.33** | **0.66** |
| V152A | A7 | 1.11 | 4.98 | 0.22 | 0.06 |
| **E154A*** | **None** | **1.96** | **2.81** | **0.70** | **0.40** |
| Q155A | Y3, T5 | 0.76 | 1.16 | 0.66 | -0.16 |
| **R157A*** | **None** | **2.14** | **1.88** | **1.14** | **0.45** |
| A158G | None | 1.63 | 1.40 | 1.16 | 0.29 |
| E161A | None | 0.94 | 0.99 | 0.94 | -0.04 |
| **T163A*** | **None** | **1.97** | **1.31** | **1.50** | **0.40** |
| **E166A*** | **None** | **1.67** | **1.24** | **1.36** | **0.31** |

**Table S4. Effect of HLA-A2 mutations on the binding of G10 TCR to HLA-A2/ GAG**

Mutated residues on the HLA-A2 heavy chain and their corresponding contact residues on the GAG peptide are listed. HLA-A2 residues not in contact with the GAG peptide and whose mutation resulted in a ΔΔG of greater than 0.3 kcal/mol (*in bold) were used for calculation of ΣΔΔG in Figure 5B. The binding data shown were averaged from at least two independent experiments and normalized to the WT K_D_, k_on_ , and k_off_ values, which were 8.3 μM, 1.8 x 10^4^ M^-1^.s^-1^ and 0.18 s^-1^, respectively.

|  | **TCR-contacting HLA-A2 residues** | **Backbone contacts** |
| --- | --- | --- |
| **1G4 TCR** | R65 | TCRβV49 - O |
|  | K68 | None |
|  | A69 | TCRβV49 - Cα |
|  | Q72 | None |
|  | A150 | TCRαQ51 - C, O |
|  | H151 | None |
|  | E154 | None |
|  | Q155 | None |
|  | **Proportion with backbone contacts** | **37.5%** |
| **A6 TCR** | E58 | None |
|  | R65 | None |
|  | K68 | TCRαW101 - C |
|  | A69 | TCRαW101 -N |
|  | A149 | TCRβR102 - C, O |
|  | A150 | TCRβG101 - O  TCRβR102 - C, O, Cα, N |
|  | A158 | None |
|  | E166 | None |
|  | R170 | None |
|  | **Proportion with backbone contacts** | **44.4 %** |
| **JM22 TCR** | R65 | None |
|  | K68 | TCRβD56 - C |
|  | Q72 | None |
|  | R75 | None |
|  | A149 | TCRβY101 - C |
|  | A150 | TCRβR98 - C, O  TCRβY101 - Cα, O |
|  | H151 | TCRβR98 - C, O |
|  | E154 | TCRαS31 - C, O |
|  | **Proportion with backbone contacts** | **62.5%** |

**Table S5. TCR contacts with HLA-A2 backbone**

TCR (1G4, A6 and JM22) contacting HLA-A2 residues were inspected to determine whether their backbone atoms were contacted by the TCR. The TCR residues involved are listed together with the corresponding MHC backbone atoms in contact. The percentage of HLA-A2 contact residues where backbone atoms contribute TCR contacts are shown.
